# Supplementary material for: Applications of Spatial Transcriptomics in Veterinary Medicine: A Scoping Review of Research, Diagnostics, and Treatment Strategies
Source: Int J Mol Sci. 2025 Jun 26;26(13):6163. doi: 10.3390/ijms26136163 (PMC12249720; doi:10.3390/ijms26136163)
Supplement: Supplementary file 1 [file ijms-26-06163-s001.zip › Supplementary File 1_6-25-25clean.pdf]

1. Study Selection and Screening Summary

1.1 Information Sources

We conducted a comprehensive literature search using PubMed, targeting studies published between 2016 and early 2025 with the most updated search string database verification conducted on February 7, 2025. The search window was selected based on the publication of the seminal paper in 2016 [1], which first introduced ST as a defined methodology for gene expression profiling. This section further describes the search strategy, data charting, and data analysis used for this scoping review.

1.2 Search Strategy

The search strategy was designed to identify studies applying ST in the context of disease diagnosis, discovery, and treatment across human and veterinary medicine. We searched PubMed using a comprehensive Boolean strategy that combined disease terms and molecular profiling terms with ST terminology to capture applications of the technology to health. The search was limited to peer-reviewed articles published in English between 2016 and early 2025 (Table S1 doi:**10.5281/zenodo.15717108**), using the following search string:

((((((((((((((((((((pathology) OR (patholog)) OR (diagnosis)) OR (pathophysiology)) OR (histopathology)) OR ("clinical pathology")) OR ("immune response"))) OR (disease*)) OR (health*)) OR (therap*)) OR (clinical*)) OR (treatment*)) OR (discover*)) OR ("in vivo")) OR ("ex vivo")) OR (model*)) OR (biomark*)) OR (medicine)) OR (microenvironment)) OR (model*)) AND (((((((((("spatial transcriptomics") OR ("spatial transcriptomic")) OR ("Transcriptomic mapping")) OR ("spatially resolved RNA sequencing")) OR ("spatially resolved transcriptomics")) OR ("gene expression mapping")) OR ("spatial transcriptome profiling")) OR ("spatial molecular profiling")) OR ("tissue-based transcriptomics")) OR ("topographical transcriptomics"))) AND (2016:2025[pdat])) AND (transcript*)*

To ensure comprehensive coverage, the search string uses a broad combination of terms across two major conceptual clusters:

- Disease and biomedical context: "pathology" OR "diagnosis" OR "pathophysiology" OR "histopathology" OR "clinical pathology" OR "immune response" OR "disease" OR "health" OR "therapy" OR "treatment" OR "biomarker" OR "medicine" OR "microenvironment" OR "clinical" OR "in vivo" OR "ex vivo" OR "model"
- Spatial transcriptomic technologies: "spatial transcriptomics" OR "spatial transcriptomic" OR "spatially resolved RNA sequencing" OR "transcriptomic mapping" OR "gene expression mapping" OR "spatial transcriptome profiling" OR "spatial molecular profiling" OR "tissue-based transcriptomics" OR "topographical transcriptomics"

We filtered results to include the keyword stem *"transcript"**. This approach allowed us to capture both biological applications and methodological advancements involving ST technologies across disciplines.

We filtered results to include the keyword stem "transcript", allowing for the comprehensive capture of both biological applications and methodological advancements involving ST across disciplines.

The complete Boolean search string was developed a priori by integrating disease-related biomedical terms with ST terminology, as outlined above. After executing the final search in PubMed, we conducted a pilot screening of the first 100 articles to assess relevance and alignment with the review objectives. The majority of retrieved studies were directly related to disease-focused applications of ST in human and veterinary contexts, confirming the conceptual sensitivity and adequacy of the search strategy. As only a minimal number of irrelevant articles (e.g., purely technical studies) were identified, no further refinement of the search string was necessary. Although screening was performed by a single reviewer, this process was guided by established methodological recommendations.

After conducting our database search, we defined a structured approach for applying inclusion and exclusion criteria described in Table 1 to our search results to select sources of evidence for the scoping review.

1.3 Selection of Sources of Evidence

Following database retrieval, all citations were imported into Rayyan AI for manual deduplication and screening. Titles and abstracts were independently reviewed by one reviewer using predefined inclusion and exclusion criteria based on study relevance, research focus, use of ST, and application to diagnostics or treatment. During screening, custom tags were applied to facilitate thematic categorization across scoping review priorities. Articles that met initial criteria were reviewed in full-text to confirm eligibility. Reasons for exclusion at the full-text stage were documented, and included studies were exported to Zotero for organization and citation management. A total of 1,398 studies were ultimately included in the review. The selection process is summarized in a PRISMA-ScR flow diagram (Figure 1). Following the PRISMA-ScR process and completing the flow diagram represent adherence to PRISMA scoping review requirements and alignment with the Arksey and O'Malley framework (Table S1).

2.. Data Charting and Analysis

To enable structured and reproducible synthesis, a comprehensive data charting and analysis strategy was developed and applied. This process involved systematically reviewing, categorizing, and labelling critical variables from each included study to support quantitative and thematic analysis. The following subsections describe the data charting and analysis

workflow, key variables labelled, and the analytic approaches used to identify patterns and trends across ST applications in human and veterinary medicine.

2.1 Data Charting Process

Data charting was conducted in multiple stages to ensure organized review, labelling, and charting. During abstract and full-text review, articles were manually tagged and labeled within Rayyan AI using custom inclusion and exclusion criteria and priority thematic categories including species, disease, organ or tissue type, method, and analysis tool. Articles selected for inclusion were then exported and organized using Zotero reference management software. Charting of key study variables for analysis was performed in Excel spreadsheets designed with individual columns for each variable tracked. Discrepancies and unclear entries were resolved through iterative review of the original, full-text sources.

2.2 Data Items

For each included study, the following data were extracted: title, author(s), year of publication, journal, species studied, disease or organ focus, cancer subtype, ST platform used, analysis tools or software, and primary application (e.g., diagnostic, therapeutic, mechanistic). When metadata items were unclear or not reported, they were excluded from quantitative synthesis. Primary application(s) were assigned based on study aims. Each publication could be coded into more than one label per category when applicable to ensure a comprehensive review.

2.3 Synthesis of Results

Extracted data were synthesized using both quantitative and thematic approaches. Descriptive statistics were used to summarize publication trends, disease categories, ST technologies, analytical tools, and species studied. Graphs and tables were generated to visualize patterns in publication year, human versus animal model use, and application category (e.g., diagnostic, therapeutic, comparative).

Results were grouped by organ system and disease subtype, including cancer, infectious disease, metabolic and reproductive, and neurodevelopmental and psychiatric disorder. Veterinary and zoonotic disease studies were analyzed separately to highlight trends relevant to One Health and comparative medicine and pathology applications. These quantitative and thematic syntheses were integrated to identify knowledge gaps, technological trends, and areas for future research.

This approach enabled a structured and flexible synthesis of diverse evidence, consistent with the Arksey and O'Malley framework and PRISMA-ScR guidelines.

3.. Protocol Registration and Appraisal of Sources

This scoping review was not registered in a protocol database. As per PRISMA-ScR recommendations and the exploratory nature of scoping reviews, no formal critical appraisal of included studies was conducted.

References

1. Ståhl, P.L.; Salmén, F.; Vickovic, S.; Lundmark, A.; Navarro, J.F.; Magnusson, J.; Giacomello, S.; Asp, M.; Westholm, J.O.; Huss, M.; et al. Visualization and Analysis of Gene Expression in Tissue Sections by Spatial Transcriptomics. *Science (80-.).* **2016**, *353*, 78–82, doi:10.1126/science.aaf2403.
